# Discrimination between Asian populations of the parasitoid wasp *Ganaspis* cf. *brasiliensis* using a simple MALDI-TOF MS-based method for use with insects

**DOI:** 10.1093/biomethods/bpz002

**Published:** 2019-03-18

**Authors:** Michael A Reeve, M Lukas Seehausen

**Affiliations:** 1 CABI, Bakeham Lane, Egham, Surrey, UK; 2 CABI, Rue des Grillons 1, Delémont, Switzerland

**Keywords:** Matrix-assisted laser-desorption and ionisation time-of-flight mass spectroscopy, Drosophila suzukii, Ganaspis cf. brasiliensis, Biological control, Regional-biotype discrimination

## Abstract

The fruit fly *Drosophila suzukii* has recently become an invasive pest insect of significant economic impact in Europe and the USA. In contrast to other *Drosophila* species, *D. suzukii* is able to infest intact fruit by means of a saw-like ovipositor, which allows females to deposit eggs beneath the skin of the fruit. Classical biological control using the parasitoid wasp *Ganaspis* cf. *brasiliensis* is currently being researched as an environmentally sustainable option for the control of *D. suzukii*. In particular, the host specificity of this parasitoid has been assessed for populations from different regions in China and Japan. In order to study the relationship between the differences in specificity and molecular variations, we have adapted a matrix-assisted laser-desorption and ionization time-of-flight mass spectrometry (MALDI-TOF MS)-based method, originally developed for use with plant material, to discriminate between example populations of *G*. cf. *brasiliensis*. We have employed a combination of principal component analysis and blind-tested comparison between reference sample MALDI-TOF MS spectra and test sample spectra to discriminate, on the basis of the acid-soluble insect protein spectra generated, between four populations of *G.* cf. *brasiliensis* (originally collected from Tokyo and Hasuike in Japan and Dali and Ximing in China). MALDI-TOF MS analysis is able to discriminate with 100% accuracy between populations *G.* cf. *brasiliensis*. The Chinese populations were observed to be similar, but the Tokyo population is slightly different and the Hasuike population is significantly different from the other populations. The Tokyo population appears more closely related to the Chinese populations than the Hasuike population, even though both originate from Japan.

## Introduction

Now a well-established laboratory technique, matrix-assisted laser-desorption and ionisation time-of-flight mass spectrometry (MALDI-TOF MS) is a versatile and powerful tool for the analysis of protein-containing samples. In this technique, large proteins are prepared intact in the gas phase carrying predominantly a single positive charge [1] by means of the MALDI soft ionization process [2]. After acceleration in an electrical field, the time-of-flight (TOF) of such a charged protein, along a tube held at high vacuum, is proportional to the square root of the mass-over-charge ratio for the protein. As a consequence of this simple relationship, a mass spectrum can be generated for the protein components in a particular biological sample [3]. The mass spectrum of a subset of the expressed proteome is generally used for the characterization and/or identification of protein-containing biological samples. This is normally the highly expressed acid-soluble cellular proteins, including many ribosomal proteins [4]. Human clinical microbiology has been a key driver behind the development of MALDI-TOF MS sample preparation and analysis [4]. This area has been extensively reviewed by Clark *et al.* [3] along with the common methods employed for MALDI-TOF MS sample preparation. Microbiologically focussed methods have also been developed for use with yeasts [5], filamentous fungi [6, 7] and mycobacteria [8]. Insect and plant materials are, however, not particularly well suited to many of the above methods [9] and so Reeve *et al.* [10, 11] have, in response, developed a highly simplified and inexpensive method for sample preparation that has broad applicability to bacteria, fungi, insects and plants. In short, this method lyses cells by immersion (with or without maceration) in aqueous acetonitrile containing trifluoroacetic acid (TFA) to selectively extract acid-soluble proteins, with lysis and extraction carried out in the presence of near-saturated and inexpensive-grade MALDI matrix. The resulting matrix-saturated lysate, containing acid-solubilized proteins, is then dried down directly onto the MALDI-TOF MS sample plate and analysed.

Regardless of the method used for sample preparation, MALDI-TOF MS still requires relatively fresh biological material, containing proteins that have not yet undergone significant degradation. In response to this, Reeve and Buddie [12] have developed a simple and inexpensive method for the practical storage of field sample proteins for subsequent MALDI-TOF MS analysis. In this method, originally developed for use with plant material, biomass is crushed onto filter paper and dried. The dried and protein-impregnated filter paper can, if required, then be treated with aqueous alcohol followed by drying, in order to inactivate any potential microorganisms of concern. After dry storage, proteins are extracted from the paper using the above solution containing acetonitrile, TFA, water and inexpensive-grade MALDI-TOF MS matrix near to saturation.

The fruit fly *Drosophila suzukii* (Matsumura) (Diptera: Drosophilidae), commonly referred to as spotted-wing Drosophila, originates from Southeast Asia, and has recently become an invasive pest insect of significant economic impact in Europe and the USA [13–15]. In contrast to other *Drosophila* species that only infest damaged and rotting fruit, *D. suzukii* is able to infest intact fruit from the early ripening stage [16]. Female *D. suzukii* accomplish this by means of a saw-like ovipositor, which allows them to deposit eggs beneath the skin of intact fruit [17]. Crop damage ensues as the resulting larvae hatch and develop inside the fruit. One environmentally sustainable option for the area-wide control of *D. suzukii* is biological control [18], in which natural enemies are sought to parasitize or predate the target species. Classical biological control (importation) uses the managed introduction and establishment of the natural enemy from the native range of the target species for control. Other biological control options include augmentation, where natural enemies are released to augment already existing populations, and conservation biological control, where environmental interventions are made in order to establish and/or maintain natural enemy populations within a given ecosystem. Among the most widely employed biological control agents are parasitoids, insects that lay their eggs on or inside their host, on which their developing larvae subsequently feed. These are generally wasps or flies, often with a very narrow host range [19]. Classical biological control using parasitoids is a well-characterized approach with a long history and, in the case of *D. suzukii*, such control may be possible using the parasitoid wasp *Ganaspis* cf. *brasiliensis* (Ihering) (Hymenoptera: Figitidae), for which a number of different populations from Asia are available [20]. The taxonomic status of this parasitoid is however unclear, and likely comprises a complex of cryptic species with different distributions [21, 22]. Variations in host specificity [21, 23] and parasitoid behaviour (M.L. Seehausen, unpublished results) of the different populations further suggest the existence of regional biotypes or cryptic species. The genetic inheritance of parasitoid behaviour and especially host specificity is an important criterion for the selection of the most suitable biological control agent.

With this in mind, one of our goals is to provide a rapid and inexpensive method for characterizing differences between regional (and other) biotypes of biological control agents and target insects (or plants) in order to optimize their matching, thereby increasing both the efficiency and the efficacy of biological control. Of the available techniques for characterizing inter- and intra-species differences, MALDI-TOF MS is a promising candidate, being both rapid and very inexpensive in terms of reagent usage and time required for sample processing.

In the current article, we have adapted the method of Reeve and Buddie for use with insect material and have used this to discriminate between four examples of *G.* cf. *brasiliensis* populations, a potential classical biological control agent against the invasive and economically damaging pest insect *D. suzukii*.

## Materials and methods

### Parasitoid collection and propagation

The different populations of *G.* cf. *brasiliensis* used in this study were originally obtained by collecting infested fresh fruits in Tokyo and Hasuike in Japan, and Dali and Ximing in China [20]. The parasitoids were then reared on *D. suzukii* larvae feeding in blueberries in the quarantine facilities of CABI in Delémont, Switzerland, as described in more detail by Girod *et al.* [23, 24].

### Reagents and paper

Ethanol ≥99.8%, ≥98% (TLC-grade) α-cyano-4-hydroxycinnamic acid (HCCA) matrix, LC–MS-grade acetonitrile, 99% ReagentPlus^®^-grade TFA, mass-spectrometry grade [50% (v/v) acetonitrile, 2.5% (v/v) TFA and 47.5% (v/v) water] and Whatman^®^ qualitative filter paper, Grade 3 (90 mm circles), were purchased from Sigma (Gillingham, UK). CHROMASOLV^TM^ LC–MS-grade water was purchased from Fluka (Loughborough, UK).

### Sample preparation

Single adult male *G.* cf. *brasiliensis* parasitoids were collected into ethanol and placed on roughly 12  × 4 mm strips of Whatman filter paper, Grade 3. The paper was then folded lengthways, wrapped in cling film and gently tapped over the whole surface using a hammer against a solid surface to transfer insect proteins to the paper. The cling film was then removed, and the paper was unfolded and allowed to air dry for 1 h. The dried and protein-impregnated paper was placed inside a capped 1.5 ml Eppendorf tube, which was stored at 4°C until protein extraction. For Method 1, proteins were extracted by immersing the paper in 200 µl of [11 mg/ml HCCA matrix in 65% (v/v) acetonitrile, 2.5% (v/v) TFA and 32.5% (v/v) water] (referred to as Solution 1 in the following), capping the tube, briefly vortexing, soaking for 10 min and vortexing again. One microlitre of the supernatant was then pipetted onto the Bruker sample plate, air dried and loaded into the spectrometer. For Method 2, 10 µl of the protein-containing Solution 1 from Method 1 was diluted with 40 µl of Solution 1. One microlitre of this dilution was pipetted onto the Bruker sample plate and air dried, followed by application of a further 1 µl of this dilution, air drying and loading into the spectrometer. For each of the two above-described methods, eight paper-only negative controls were carried out using 6-mm diameter Whatman filter paper, grade 3 discs.

### MS

MS covering the range from 2  to 20 kDa was carried out using a Bruker Microflex LT linear-mode instrument running the MALDI Biotyper 4.0 applications (Bruker Daltonik, Bremen, Germany), using a 60 Hz frequency and 3 ns pulse duration nitrogen laser (70 µJ, with maximum output 225 µJ), with a wavelength of 337 nm and spot size of 100 µm, with 240 laser shots per sample. The laser settings were Global Attenuator Offset (0%), Attenuator Offset (25%) and Attenuator Range (30%), and the ion source voltage was 19.98 kV. Bruker MBT Biotarget 96 plates (Bruker ref. 1840375) were used for all samples in this study. Calibration was carried out using the manufacturer’s ‘BTS’ controls (*Eshcerichia coli* proteins supplemented with ribonuclease A and myoglobin), using peaks with masses at 3637.8; 5096.8; 5381.4; 6255.4; 7274.5; 10 300.2; 13 683.2 and 16 952.3 for calibration according to the manufacturer’s instructions. Spectra were acquired using MALDI Biotyper RTC Version 4.0 (Build 19) using the manufacturer’s standard settings (Centroid peak-detection algorithm and TopHat baseline subtraction). Database entries were made as single-spectra Main Spectra (MSPs) using the Bruker Online Client software suite (Version 4.0.19, Bruker Daltonik, Bremen, Germany), again using the manufacturer’s standard settings. For spectral comparisons, Bruker identification scores were derived using the standard Bruker algorithm. This first converts raw mass spectra into peak lists, which are then compared between spectra. Three separate values are computed: the number of peaks in the reference spectrum that have a closely matching partner in the test spectrum (value range 0–1), the number of peaks in the test spectrum that have a closely matching partner in the reference spectrum (value range 0–1) and the peak height symmetry of the matching peaks (value range 0–1). The above three values are multiplied together and normalized to 1000, and the base-10 logarithm is then taken to give the final Bruker score (range 0–3). Bruker scores of scores between 2.3 and 3.0 indicate very close relatedness, scores between 2.0 and 2.3 indicate close relatedness and scores <1.7 indicate low relatedness.

### Spectral comparison

Four ‘reference’ sample preparations were carried out as indicated for each of the four populations, from which a database of 16 reference spectra was generated for each method. For blind testing, randomized and numbered tubes were supplied containing six samples of each population, giving a panel of 24 ‘test’ samples for each method. For spectral comparison, all test samples were compared against the database of reference spectra, and Bruker identification scores were generated as described above. These were then averaged for each of the reference sample population, and the highest of these average values was used as the identification call for each test sample.

### Statistical analysis

Spectral comparisons using principal component analysis (PCA) were carried out using the Bruker On-line Client software. PC1 versus PC2 ordination plot were generated, PCA was unsupervised, and all peaks were weighted equally. Differences in Bruker scores between test spectra were analysed for each reference spectrum of a parasitoid population separately using one-way analyses of variance, with the scores as dependent and parasitoid population as independent variable. In all cases, the residuals sufficed the assumptions of normal distribution and homogeneity of variance, as assessed graphically.

## Data availability 

Original spectral data held on the Bruker Microflex PC is available on request.

## Results

All eight paper-only controls for sample preparation Method 1 (described in the Methods section) gave no peaks (Supplementary data, Fig. S1) and seven out of eight spectra for Method 2 gave no peaks, with the remaining negative control showing two small peaks (Supplementary data, Fig. S2, shown with the same scaling as the BTS positive controls), which likely result from the double layer of matrix and the relatively high laser power used.

**Figure 1:**
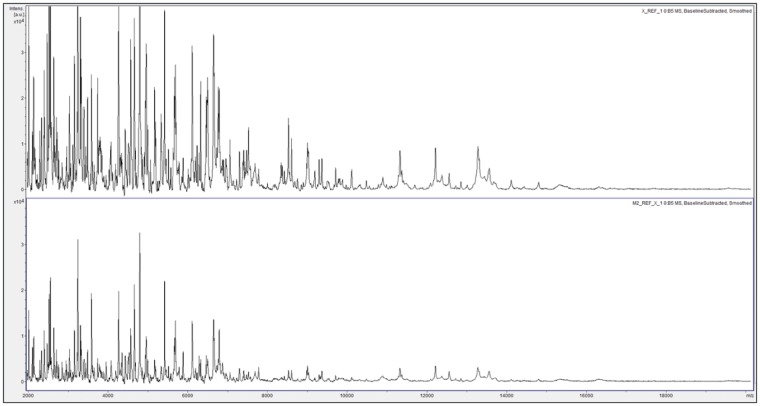
MALDI-TOF MS spectra of acid-soluble insect proteins for an example population of *Ganaspis* cf. *brasiliensis* (Ximing, reference Sample 1) obtained using Method 1 (top), and Method 2 (bottom) as described in the Materials and Methods section. Spectra are shown baseline-subtracted, smoothed, with common scaling on the *y*-axis (−2000 to 40 000 Bruker Intensity Units), and covering the mass range from 2 to 20 kDa (with *x*-axis scale increments of 2 kDa).

**Figure 2:**
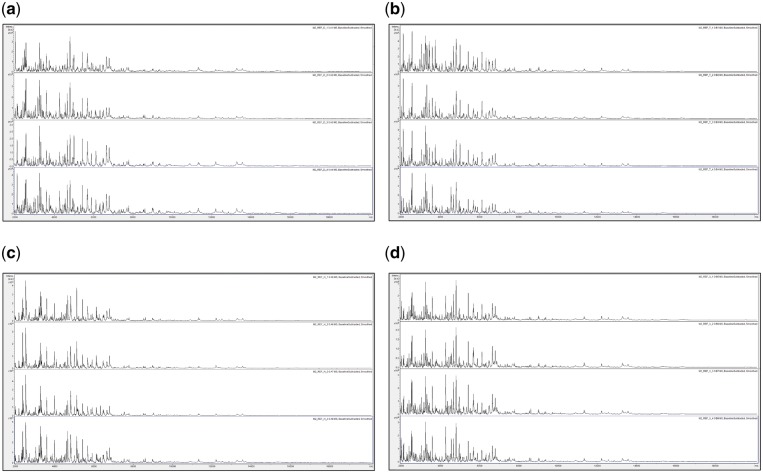
Quadruplicate reference MALDI-TOF MS spectra of acid-soluble insect proteins from *Ganaspis* cf. *brasiliensis* populations originating from Dali (a), Hasuike (b), Tokyo (c) and Ximing (d) obtained using Method 2 as described in the Materials and Methods section. Spectra are shown baseline-subtracted, smoothed, with *y*-axis autoscaling and covering the mass range from 2  to 20 kDa (with *x*-axis scale increments of 2 kDa).

Using Method 1, the overall accuracy in blind testing was 19.5 correct identifications from 24 tests (81%, Supplementary data, Table S1), which is somewhat lower than we have generally observed in similar studies with plant materials [11]. While observing the real-time data acquisition, it was apparent that very strong signals were generated by many of the laser shots, often leading to saturation of the detector and ‘clipping’ of the resulting peaks. Reasoning that this will have an effect on the distribution of peak heights across the mass range (which are taken into account by the Bruker algorithm when comparing spectra) as well as the accuracy of mass assignment (because this is based upon peak centroids), we therefore carried out 5-fold dilutions in Solution 1 (described in the Materials and Methods section) of the samples reported in Supplementary data, Table S1. In order to increase the density of matrix crystals (and therefore the spot-to-spot reproducibility of the MALDI process), these were also deposited as two consecutive 1 µl droplets, with drying in between (Method 2). This resulted in a reduced signal strength compared to Method 1 (Fig. 1) as well as high peak richness and an excellent replicate-to-replicate reproducibility of the MALDI-TOF MS spectra obtained for each parasitoid population (Fig. 2).

The average Bruker scores obtained from Method 2 resulted in an overall repeat-testing accuracy of 24 correct identifications from 24 tests (100%, Table 1), which is comparable to what we have generally observed in similar studies with plant materials [11]. This result, combined with the high-quality MALDI-TOF MS spectra illustrated in Figure 2, demonstrates that Method 2 gives informative and very reproducible spectra that are capable of very high-level discrimination between insects, down to regional populations of what is described as the same species.

**Table 1:** Average Bruker score against quadruplicate reference spectra from populations of *Ganaspis* cf. *brasiliensis* (originally collected from Tokyo and Hasuike in Japan, and Dali and Ximing in China) using Method 2 (described in the Materials and Methods section)

	Average Bruker score against quadruplicate reference spectra for populations from					
Repeat test number	Dali	Hasuike	Tokyo	Ximing	Identification call	Correct or incorrect?
1	1.788	2.376	1.742	1.826	Hasuike	Correct
2	2.499	1.735	2.409	2.419	Dali	Correct
3	2.439	1.830	2.575	2.375	Tokyo	Correct
4	2.450	1.693	2.247	2.597	Ximing	Correct
5	1.696	2.384	1.672	1.651	Hasuike	Correct
6	1.784	2.156	1.819	1.762	Hasuike	Correct
7	2.415	1.757	2.287	2.423	Ximing	Correct
8	2.476	1.595	2.406	2.373	Dali	Correct
9	1.844	2.509	1.887	1.810	Hasuike	Correct
10	2.506	1.781	2.373	2.436	Dali	Correct
11	2.500	1.814	2.280	2.514	Ximing	Correct
12	2.528	1.714	2.424	2.419	Dali	Correct
13	2.437	1.735	2.301	2.529	Ximing	Correct
14	1.843	2.269	1.800	1.828	Hasuike	Correct
15	2.385	1.856	2.565	2.377	Tokyo	Correct
16	2.302	1.641	2.461	2.236	Tokyo	Correct
17	2.365	1.800	2.510	2.266	Tokyo	Correct
18	2.452	1.725	2.293	2.543	Ximing	Correct
19	2.491	1.826	2.339	2.561	Ximing	Correct
20	1.795	2.424	1.762	1.799	Hasuike	Correct
21	2.471	1.769	2.284	2.467	Dali	Correct
22	2.379	1.735	2.404	2.290	Tokyo	Correct
23	2.524	1.779	2.414	2.449	Dali	Correct
24	2.461	1.771	2.617	2.427	Tokyo	Correct

The highest average Bruker score is given as the identification call in the penultimate column, and the accuracy of this is shown the final column.

**Figure 3:**
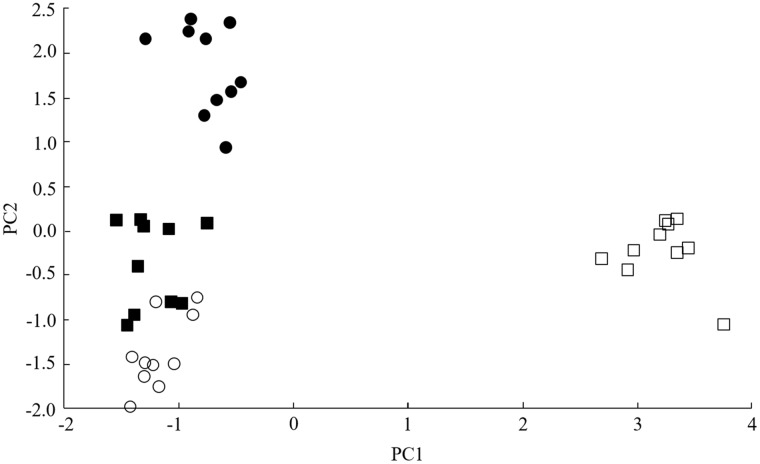
PCA ordination plot for the 16 reference sample MALDI-TOF MS spectra and the 24 unblinded test sample spectra of acid-soluble insect proteins from the different *Ganaspis* cf. *brasiliensis* populations originally collected from (filled square) Dali, (open square) Hasuike, (filled circle) Tokyo and (open circle) Ximing. PCA was unsupervised, and all peaks were weighted equally.

**Figure 4:**
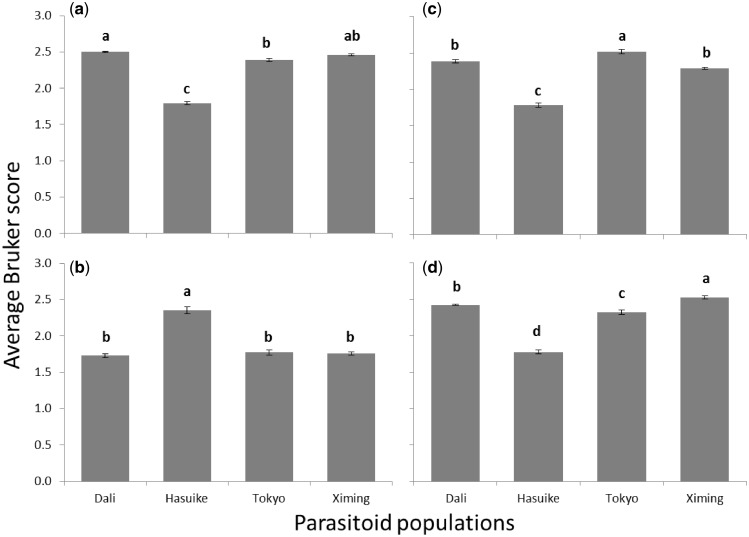
Average (±SE) Bruker score values from Method 2 test spectra for each *Ganaspis* cf. *brasiliensis* population compared against reference spectra for populations from (A) Dali, (B) Hasuike, (C) Tokyo and (D) Ximing. Bars with the same lower case letters are not significantly different at α = 0.05 according to Tukey’s HSD test.

In order to investigate the relatedness between the various samples, the spectra used to derive Table 1 were used for PCA. The ordination plot (Fig. 3) shows well separated grouping of the spectra for the Hasuike population (open square) and, to a lesser extent, separated grouping of the spectra for the Tokyo population (filled circle), with the spectra for the Dali (filled circle) and Ximing (open circle) populations forming an overlapping grouping in the ordination plot. While the Hasuike population is well separated in the ordination plot, the Tokyo populations (also originating from Japan) appears to be much more closely related to the Chinese populations from Dali and Ximing than the (Japanese) Hasuike population. Knowing the identity of the 24 repeat-test samples, we organized the data from Table 1 according to the different populations of the test samples (Supplementary data, Table S2). From this, we calculated average values, for each set of six population test samples, of the average Bruker scores against the site-specific quadruplicate reference spectra. These average values, with error bars indicating one standard error either side of the mean, are shown in Figure 4.

A significant effect of the affiliation to different parasitoid populations on the Bruker score was found for all reference spectra: Dali (*F*_3,__20__ _= 342.29; *P* < 0.0001), Hasuike (*F*_3,__20__ _= 74.68; *P* < 0.0001), Tokyo (*F*_3,__20__ _= 164.43; *P* < 0.0001) and Ximing (*F*_3,__20__ _= 182.94; *P* < 0.0001). Figure 4 shows very high spectral similarity between the populations originating from Dali and Ximing, with a significant difference in the average Bruker scores between the two populations only when taking Ximing as the reference spectrum (Fig. 4C). In all comparisons, the Hasuike population, displays significant spectral divergence from all other populations (Fig. 4A–D) and, in two of the comparisons, the Tokyo population also shows a significant divergence from all other populations (Fig. 4C and D). This broadly agrees with the results from the PCA ordination plot shown in Figure 3, further supporting the hypothesis that the Hasuike population is somewhat unrelated to the other populations and that the Tokyo population is slightly different from the Chinese populations but is more closely related to the Chinese than to the other population (Hasuike) originating from Japan.

## Discussion

Reeve and Buddie have previously reported a simple and inexpensive method, originally developed for use with plant material, for the practical storage of field sample proteins for subsequent MALDI-TOF MS analysis [12]. In the current article, we have successfully adapted this method for use with insect material, and thereby to discriminate between example populations of the parasitoid *G.* cf. *brasiliensis*. The approach that we have used above (and in [10–12]) for high-resolution spectral comparisons between very similar analytes departs slightly from the ‘standard Bruker method’ (which was developed for, and works very well with, routine clinical identifications of bacteria and yeasts). Our reasoning for this is that the use of multiple replicates in experimental work is designed to accommodate variance of some component of what is being measured. In the case of MALDI-TOF MS, the sources of variance are: shot-to-shot laser variance (averaged over 240 shots in the Bruker), well-to-well pipetting variance, preparation-to-preparation technical variance, and sample-to-sample biological variance. In our experience, shot-to-shot laser variance and well-to-well pipetting variance have a very small impact on MALDI-TOF MS spectral variability. In sharp contrast, preparation-to-preparation technical variance and sample-to-sample biological variance can have a major impact. For the former, we have developed methods that aim to reduce technical variance [10–12] so that the major remaining source of variance is sample-to-sample biological variance. The ‘standard Bruker method’ takes a single biological sample and a single sample preparation and then averages only from multiple pipettings and multiple laser shots whereas in our approach, in order better to accommodate experimental variances, we instead use a single pipetting and a single set of laser shots but use multiple sample preparations (where possible) and multiple biological samples. In addition, an MSP derived from multiple source spectra is a statistical construct; it is not a real spectrum of any sample. In our approach, every single spectral comparison is between actual spectra from actual samples, which we believe is more appropriate for our purposes.

Using the above approach, initial blind-testing for discrimination between populations using Method 1 gave an overall accuracy somewhat lower than we have generally observed in similar studies with plant materials [11]. While observing the real-time data acquisition, it was apparent that very strong signals were being generated by many of the laser shots, often leading to saturation of the detector and ‘clipping’ of the resulting peaks. Reasoning that this will have an effect on the distribution of peak heights across the mass range (which are taken into account by the Bruker algorithm when comparing spectra) as well as the accuracy of mass assignment (because this is based upon peak centroids), we therefore developed Method 2, which also increased the density of matrix crystals (and therefore the spot-to-spot reproducibility of the MALDI process). Method 2 did indeed give reduced signal strength compared to Method 1, and resulted in appropriate peak richness and excellent replicate-to-replicate reproducibility, allowing repeat-testing results for discrimination between regional *G.* cf. *brasiliensis* populations now with an accuracy of 100%.

The wasp populations used in this study were all reared under the same laboratory conditions on *D. suzukii* larvae feeding on blueberries. Under field conditions, however, the parasitoids might attack *D. suzukii* larvae feeding on different fruits [20, 22], or even attack other host species [21–23]. This raises the question if field-collected samples from different fruits or host species would result in equally consistent identifications of populations. MALDI-TOF MS analysis is based on the relatedness of expressed acid-soluble subproteomes, which have components determined by genetics, epigenetics and the environment. While genetic determination is stable in the absence of mutation, the impacts of epigenetic and environmental changes outside of laboratory conditions (e.g. different insect diets) on insect characterization and/or identification can only be determined empirically. This is, however, beyond the scope of the present study.

We observed clearly separated PCA grouping of the spectra for the Hasuike population and separated grouping of the spectra for the Tokyo population, with the spectra for the Dali and Ximing (Chinese) populations overlapping. While the Hasuike population is well separated from all other populations in the ordination plot, the Tokyo population (also originating from Japan) appears to be more closely related to the Chinese populations than the Hasuike population. This conclusion is also supported by the spectral comparison data. Interestingly, from the four tested populations, Hasuike is the only one that originates from a site in a temperate climate zone (a ski resort in the Nagano Prefecture), while the other three are all from a subtropical climate zone [20]. The average Bruker score between test samples and their cognate reference samples from Table 1 is 2.476 and the average Bruker score between: Hasuike test samples and non-cognate reference samples, combined with (in the reverse direction) non-cognate test samples and Hasuike reference samples, is 1.768. When used for comparison between an unknown test sample spectrum and a database of known reference sample spectra, the guideline interpretations for the Bruker scores used for ‘identification’ in this manner are: 2.300–3.000, ‘highly-probable species-level identification’; 2.000–2.299, ‘secure genus-level identification and probable species-level identification’; 1.700–1.999, ‘probable genus-level identification’; and 0.000–1.699, ‘no reliable identification’. If used for comparison on this basis, then the above average Bruker score of 2.476 would comfortably indicate ‘highly-probable species-level identification’ but the score of 1.768 would only indicate ‘probable genus-level identification’ for the difference between the Hasuike population and the remaining ones, which might suggest that the Hasuike population is possibly the same genus but a different species compared with the remaining populations of *G*. cf*. brasiliensis*. Crossing experiments between the Hasuike population and others should be carried out to confirm this result. However, given that the improved spectral comparison of the populations allowed for a 100% accurate identification of populations from different regions, one may assume a distinct molecular (and/or genetic) differentiation between all populations, which at least indicates the existence of regional biotypes. These tentative conclusions must, however, be treated with caution because, in contrast to DNA sequencing-based approaches, MALDI-TOF MS analysis is not a comparably linear technique because unit changes in DNA sequence (a common measure of biological difference) do not necessarily lead to unit changes in protein molecular weight. By referring to the genetic code [25], at one extreme, mutation of the protein-encoding in-frame sequence CTT to ATT will convert a leucine residue to an isoleucine residue, resulting in no change in protein molecular weight. At the other extreme, mutation of the protein-encoding in-frame sequence GGG to TGG will convert a glycine residue to a tryptophan residue, resulting in a protein molecular weight change of some 129 Da.

In conclusion, we have successfully adapted the method of Reeve and Buddie, originally developed using plant material, for the analysis of insects. We have employed a combination of PCA and blind-tested comparison between reference sample MALDI-TOF MS spectra and test sample spectra to discriminate, on the basis of the acid-soluble insect-protein spectra generated, between four populations of the potential biological control agent *G.* cf. *brasiliensis* (originally collected from Tokyo and Hasuike in Japan, and Dali and Ximing in China), for which MALDI-TOF MS analysis is able to discriminate with 100% accuracy between populations. The Chinese populations were observed to be very similar, but significant differences were found between the Hasuike population and all others, as well as, in some cases, between the Chinese populations and the Tokyo population. The Tokyo population appears more closely related to the Chinese populations than the Hasuike population even though both originate from Japan.

## Supplementary Material

Supplementary Data
