# Supplementary material for: Discrimination between Asian populations of the parasitoid wasp Ganaspis cf. brasiliensis using a simple MALDI-TOF MS-based method for use with insects
Source: Biol Methods Protoc. 2019 Mar 18;4(1):bpz002. doi: 10.1093/biomethods/bpz002 (PMC7206448; doi:10.1093/biomethods/bpz002)
Supplement: Supplementary Data [file bpz002_supp.pdf]

Supplementary material

Table S1 Average Bruker score against quadruplicate reference spectra from populations of *Ganaspis* cf. *brasiliensis* (originally collected from Tokyo and Hasuike in Japan, and Dali and Ximing in China) using Method 1 (described in the methods section). The highest average Bruker score is given as the identification call in the penultimate column, and the accuracy of this after unblinding the data is shown the final column.

	Average Bruker score against quadruplicate reference spectra for populations from					
Blind-test number	Dali	Hasuike	Tokyo	Ximing	Identification call	Correct or incorrect?
1	1.852	2.345	1.725	1.608	Hasuike	Correct
2	2.381	1.711	2.381	2.333	Dali/Tokyo	Partially correct (Dali)
3	2.406	1.762	2.360	2.117	Dali	Incorrect (Tokyo)
4	2.347	1.611	2.164	2.535	Ximing	Correct
5	1.790	2.242	1.583	1.592	Hasuike	Correct
6	1.731	2.191	1.727	1.607	Hasuike	Correct
7	2.375	1.663	2.312	2.445	Ximing	Correct
8	2.466	1.738	2.215	2.205	Dali	Correct
9	1.733	2.424	1.746	1.679	Hasuike	Correct
10	2.435	1.749	2.174	2.175	Dali	Correct
11	2.337	1.603	2.191	2.466	Ximing	Correct
12	2.523	1.688	2.321	2.375	Dali	Correct
13	2.356	1.682	1.992	2.378	Ximing	Correct
14	1.813	2.294	1.803	1.560	Hasuike	Correct
15	2.210	1.693	2.410	2.044	Tokyo	Correct
16	2.257	1.776	2.329	2.016	Tokyo	Correct
17	2.232	1.634	2.073	2.007	Dali	Incorrect (Tokyo)
18	2.394	1.768	2.200	2.383	Dali	Incorrect (Ximing)
19	2.379	1.742	2.129	2.316	Dali	Incorrect (Ximing)
20	1.805	2.437	1.785	1.763	Hasuike	Correct
21	2.405	1.867	2.255	2.387	Dali	Correct
22	2.304	1.795	2.439	2.148	Tokyo	Correct
23	2.284	1.475	2.256	2.245	Dali	Correct
24	2.341	1.801	2.448	2.166	Tokyo	Correct

Table S2 Data from Table 1 reorganised according to the populations of the test samples.

		Average Bruker score against quadruplicate reference spectra for populations from			
Correct identity	Repeat-test number	Dali	Hasuike	Tokyo	Ximing
Dali	2	2.499	1.735	2.409	2.419
Dali	12	2.476	1.595	2.406	2.373
Dali	14	2.506	1.781	2.373	2.436
Dali	17	2.528	1.714	2.424	2.419
Dali	27	2.471	1.769	2.284	2.467
Dali	29	2.524	1.779	2.414	2.449
Hasuike	1	1.788	2.376	1.742	1.826
Hasuike	9	1.696	2.384	1.672	1.651
Hasuike	10	1.784	2.156	1.819	1.762
Hasuike	13	1.844	2.509	1.887	1.810
Hasuike	20	1.843	2.269	1.800	1.828
Hasuike	26	1.795	2.424	1.762	1.799
Tokyo	3	2.439	1.830	2.575	2.375
Tokyo	21	2.385	1.856	2.565	2.377

Tokyo	22	2.302	1.641	2.461	2.236
Tokyo	23	2.365	1.800	2.510	2.266
Tokyo	28	2.379	1.735	2.404	2.290
Tokyo	30	2.461	1.771	2.617	2.427
Ximing	8	2.450	1.693	2.247	2.597
Ximing	11	2.415	1.757	2.287	2.423
Ximing	15	2.500	1.814	2.280	2.514
Ximing	19	2.437	1.735	2.301	2.529
Ximing	24	2.452	1.725	2.293	2.543
Ximing	25	2.491	1.826	2.339	2.561

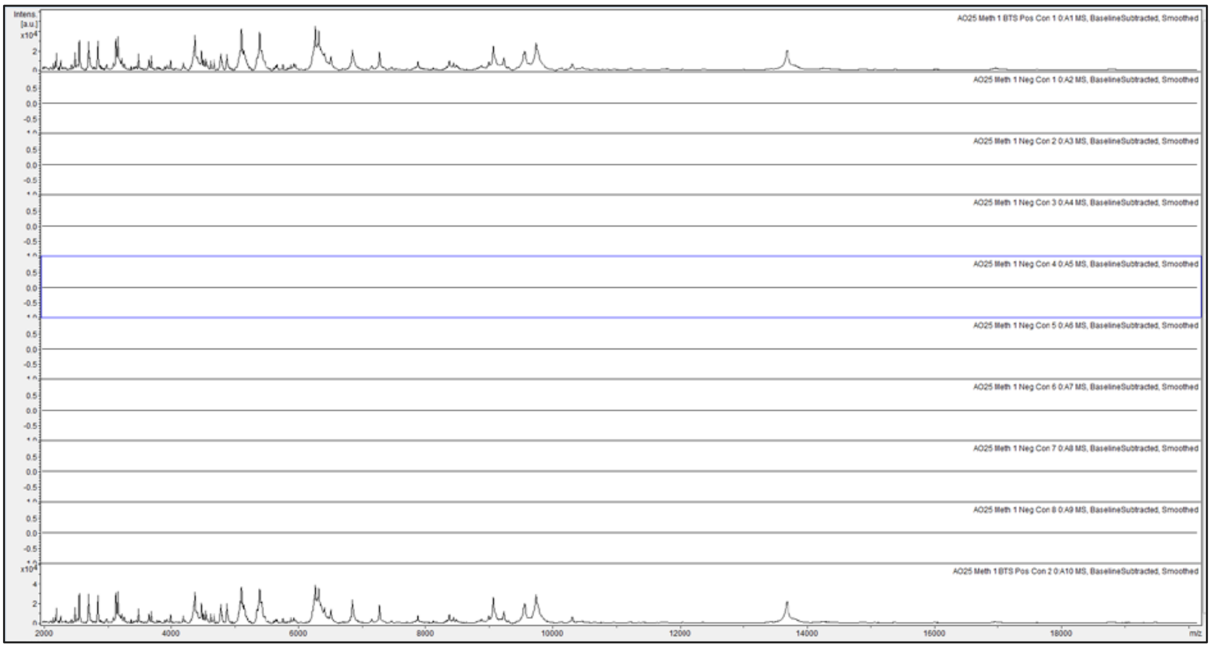

Figure S1 Method 1 MALDI-TOF MS control spectra with, from top to bottom, BTS positive control #1, negative controls 1-8, and BTS positive control #2. Spectra are shown baseline-subtracted, smoothed, with y-axis scaling between -2,000 and 60,000 Bruker intensity units, and covering the mass range 2 kDa to 20 kDa (with x-axis scale increments of 2 kDa).

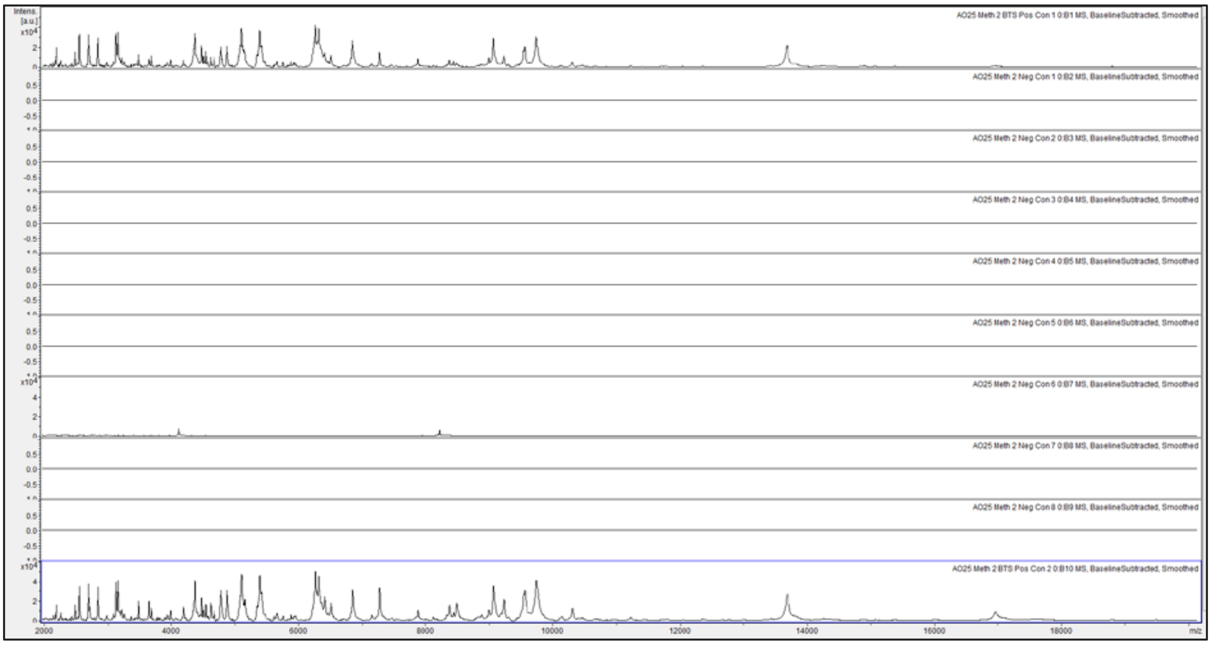

Figure S2 Method 2 MALDI-TOF MS control spectra with, from top to bottom, BTS positive control #1, negative controls 1-8, and BTS positive control #2. Spectra are shown baseline-subtracted, smoothed, with y-axis scaling between -2,000 and 60,000 Bruker intensity units, and covering the mass range 2 kDa to 20 kDa (with x-axis scale increments of 2 kDa). Two small peaks were obtained for negative control #6 using Method 2.
